# 

**Published:** 1851-08

**Authors:** 


### THE WESTERN JOURNAL OF MEDICINE AND SURGERY:

EDITED BY
LUNSFORD P. YANDELL, M. D.,
PROFESSOR OF PHYSIOLOGY AND PATHOLOGICAL ANATOMY IN THE UNIVERSITY OF LOUISVILLE,
AND
THEODORE S. BELL, M. D.

CXL.—AUGUST, 1851.

THIRD SERIES.
VOL. VIII......NO. II.

LOUISVILLE, KY:
PUBLISHED MONTHLY BY PRENTICE & WEISSINGER,
PROPRIETORS AND PRINTERS.
1851.

[POSTAGE 6¼ CENTS.]

